# Bromelain-based enzymatic debridement as a treatment of choice in high-risk patient with deep facial burns, a case report

**DOI:** 10.1016/j.ijscr.2020.04.052

**Published:** 2020-05-11

**Authors:** Nikola Ferancikova, Peter Bukovcan, Nina Sarkozyova, Jana Dragunova, Valeria Cucorova, Jan Koller

**Affiliations:** a Teaching Department of Burns and Reconstructive Surgery, Medical Faculty of Comenius University & University Hospital Bratislava, Ruzinovska 6 (Ružinovská), 82101, Bratislava, Slovakia; b Central Tissue Bank at Department of Burns and Reconstructive Surgery, University Hospital Bratislava, Pazitkova 4 (Pažítková), 82101, Bratislava, Slovakia

**Keywords:** Facial burns, Deep burns, Enzymatic debridement, Inhalation injury, Case report

## Abstract

**Introduction:**

Deep facial burns are often combined with inhalation injury that could lead to patient destabilization. Accurate timing of surgical debridement of deep burns in a critical patient is the real medical art. Especially in patients with deep burned face and hands, in whom early debridement promises better functional and aesthetic results.

**Case presentation:**

A fifty-three-year-old woman sustained burns of 16% TBSA including face area. The treatment of the burn injury was complicated by severe inhalation trauma, which led to patient destabilization shortly after admission. Standard surgical debridement was risky at the time. We used a new enzymatic agent for early burn eschar necrolysis instead. All the debrided areas were temporarily covered with porcine xenografts. The facial burns healed spontaneously without the need for a skin transplant. Definitive surgery treatment of full-thickness burns was postponed until the patient´s stabilization.

**Discussion:**

The new enzymatic debridement is minimally invasive and can be applied bedside without the need for general anesthesia. All advantages of the new enzymatic debridement had led to extend its use at the face area, although it was not tested in this area during pre-registration studies. Especially in facial area high selectivity and significant reduction of skin grafting expect a better aesthetic and functional outcome.

**Conclusion:**

Bromelain-based enzymatic debridement proved to be safe and effective on the face in a very high-risk patient with unstable circulation and severe inhalation injury as an alternative to tangential excision.

## Highlights

• Treatment of deep facial burns is one of the most challenging problems in burn care.
• Burn eschar increases the risk of infection and hypertrophic scar formation.
• Deep facial burns are often associated with inhalation injury.
• A new bromelain-based proteolytic enzyme is available for deep burn debridement.
• The bromelain-based agent was not tested in patients with unstable vital functions.

## 1 Introduction

Deep burns are characterized by the presence of necrotic tissues that strongly adhere to the wound bed. Burn eschar significantly increases the risk of early and late complications, such as infection and hypertrophic scar formation. Therefore, early burn eschar removal became a cornerstone in deep burns therapy. The current method of choice in the debridement of deep burns is tangential necrosectomy. Tangential necrosectomy is a quick and effective surgical method of eschar removal, however, it is associated with severe blood loss and removal of quite large amounts of healthy tissues as well. The negative consequences of surgical necrosectomy led to the development of less invasive methods of burns debridement – enzymatic necrolysis. Currently, a new enzymatic, bromelain-based plant proteolytic enzyme is available for deep burn debridement. Extensive pre-registration multicentre clinical studies did show that this method proved to be a rapid and highly selective minimal invasive modality for burn eschar removal [[1], [2], [3], [4]].

Treatment of deep burns of the facial area is one of the most challenging problems in burn care as they often result in mutilating scar formation. Deep facial burns are often associated with inhalation injury as well. We present a case of using enzymatic debridement in a patient with deep facial burns accompanied by severe inhalation injury. Although the use of the new enzymatic product was not tested at the facial area during preregistration clinical studies, several cases of using the product on the face have been reported with good results and no adverse events. [5,6]

This article has been written according to SCARE criteria [7].

## 2 Case presentation

**Fig. 1:**
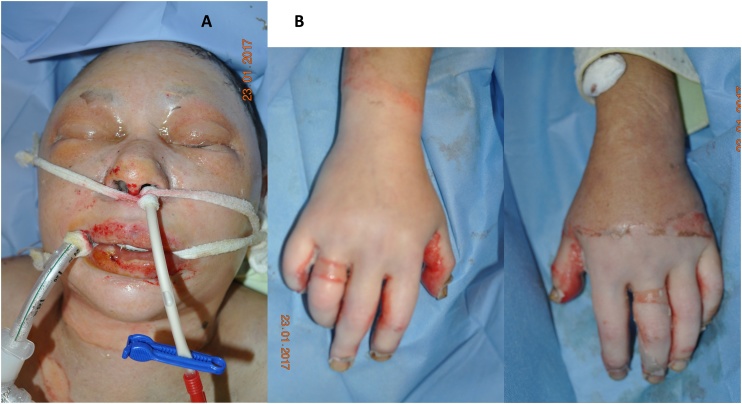
**A: Pre-debridement.** Deep facial burns clinically diagnosed as mixed deep dermal/third degree. **B: Pre-debridement.** Third degree burns at both of the hands according to clinical criteria.

A 53-year-old female patient has got burned when handling a propane-butane gas cartridge that exploded at her kitchen. The patient sustained 16% TBSAB (Total Body Surface Area Burned) to face, neck, trunk, both hands and right thigh associated with severe inhalation trauma. The burn depth was diagnosed clinically as mixed deep dermal/third degree of 12% TBSAB (third-degree burns at hands, neck and lower part of the face, total 6%) and superficial burns of 4% TBSAB (Fig. 1). Because of inhalation injury, the patient was intubated immediately at admission. There was an extensive edema of the epiglottis and a huge mass of carbon blacks in examined airways. Shortly after admission, despite adequate fluid replacement therapy, the patient´s circulation became unstable. High doses of circulatory support drug (norepinephrine) were needed for the restoration of normal blood pressure although there were not any cardiac or other internal diseases in the patient´s medical history. In that condition early surgical intervention was risky and it was replaced by enzymatic debridement.

**Fig. 2:**
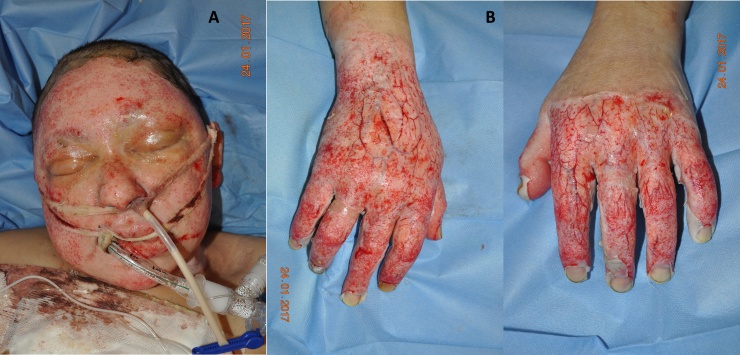
**A: Post-debridement.** A vital dermis was found at the wound bed of the whole facial area after debridement. **B: Post-debridement.** The thrombotized vessels remained in the wound beds after debridement on both hands.

The enzymatic debridement of the deep burn areas at the face, neck, both hands and trunk (total 8% TBSA), was performed 17 h post-injury using bromelain-derived proteolytic enzymes mixture gel. The procedure was accomplished according to the manufacturer´s instructions. We added just one more step, protection of the orbital area and outer ear canal with medical-grade sterile vaseline. Single debridement efficacy was 95% on the face, neck and trunk and 90% on both hands. (Fig. 2) In the high-risk patient with unstable circulation enzymatic debridement proved to be safe and effective.

All the debrided wounds were covered temporarily by cryopreserved porcine xenografts. The porcine xenografts were standardly processed in the Central Tissue Bank at Department of Burns and Reconstructive Surgery, University Hospital, Bratislava. Healing by epithelization under the xenografts of most of the wounds including face area was achieved within 5 weeks. Only 2,5% TBSA of the debrided areas on both hands and neck were full-thickness deep burns. Full-thickness burns were closed by split-thickness autografts after patient stabilization, fifteen days after the debridement. The patient was discharged from our department 5 weeks after injury with all burned areas healed.

**Fig. 3:**
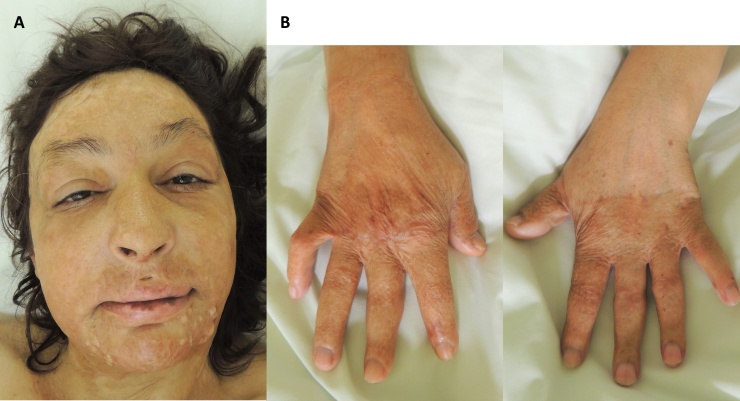
**A: Follow-up.** Inconspicuous scar formation after spontaneous healing in the facial area with few sebaceous cysts at chin. **B: Follow-up.** Appropriate scar formation of the grafted areas on both hands with scar contracture on the right hand.

We followed-up the patient for 20 months after the injury (Fig. 3). The quality of scars after spontaneous healing at the patient´s face and neck was satisfying with good aesthetical and functional outcome. Only a few sebaceous cysts formed at the patient´s chin. The scars after skin grafting at the patient´s left hand were also satisfying with full function of the hand. Only one scar contracture formed in the grafted area at the patient´s right hand, which limited the flexion of the little finger and the index finger. The patient was readmitted 20 months post-injury for the contracting scars revision. Scar contractures of the 2 above-mentioned fingers of her right hand have been released using Z-plasties. Sebaceous cysts from the chin have been removed as well. The patient was discharged after 2 days and did not come back for follow-up anymore.

## 3 Discussion

Present clinical experience with using bromelain-based agent in the treatment of deep burns did show several advantages of enzymatic debridement compared with tangential necrosectomy: shorter time to complete debridement, reducing the need for surgery, reducing the extent of excised areas, reducing the need of skin grafting [5,6,[8], [9], [10]].

There was a discrepancy between initial clinical diagnosis of the burn depth and the burn depth evaluation following the debridement using bromelain-based agent. In many cases, the burns have been more superficial than they were clinically diagnosed at patient admission. High selectivity of the bromelain-based enzymatic debridement enabled preservation of viable dermis in the wound bed after debridement of deep dermal burns. As far as parts of vital dermis remain at the wound bed, the burns can heal by spontaneous epithelialization. After the enzymatic debridement skin autografting is necessary only for the treatment of full-thickness skin loss. Our patient was clinically diagnosed with full-thickness burns at 6% of the TBSA including part of the facial area. After the enzymatic debridement, the burns of the whole facial area and most of the neck were reassessed as deep dermal, as remaining vital dermis was found at the wound bed after debridement. Skin autografting was required only in areas with full-thickness skin loss in both hands and a small part at the neck [[8], [9], [10], [11], [12]].

The advantages of the new enzymatic debridement did lead to an extension of its use at the face area, although it was not tested in this area during the pre-registration clinical studies. Especially in the facial area the high selectivity and the significant reduction of autografting did expect a better aesthetic and functional outcome. Information about using bromelain-based agent in the facial area is limited so far. The first published publication by Spanish authors demonstrated the safe use of the bromelain-based agents for deep dermal burns in the facial areas in 10 patients. There were no infectious complications of the eye region and no need for autografting after enzymatic debridement [5]. A unique comparative study using bromelain-based enzymatic debridement for deep facial burns was published by German authors. The German study confirmed the safe and beneficial use of enzymatic debridement in the facial area. In addition, the authors concluded that the quality of the scars was found significantly better in patients undergoing the enzymatic debridement compared to the patients treated by surgical necrosectomy [6].

The previous studies have confirmed the safe and effective use of the Nexobrid™ in burned patients. But there are also some limitations of the use of the enzymatic debriding agent. An absolute limitation of the enzymatic debridement is its exclusive use in the adult population. The usage of enzymatic debridement in the pediatric population is not approved yet. Naturally, the bromelain-based enzymatic agent should not be administered to people with known allergies to bromelain, pineapple or any of the Nexobrid™ ingredients. Also the cross-sensitivity between bromelain and papain, latex proteins, bee venom and olive pollen has been reported in the literature. The enzymatic debridement is not recommended in patients with chemical burns as potential interactions of the NexoBrid™ with acids and lyes cannot be excluded. The same applies to wounds contaminated with radioactive and other hazardous substances. The NexoBrid™ is not recommended also in the treatment of penetrating burns, which could expose foreign material (eg implants, pacemakers, shunts) or important anatomical structures after debriding [3,13].

The bromelain-based enzymatic debridement should be performed within 72 h after the injury, as the efficacy of enzymatic debridement is reduced in case of burns of an earlier date. [3] The enzymatic debridement procedure is accompanied by pain of varying intensity. Some patients experience severe pain, others burning, tingling or itching. The analgesics should be administered to the patient at least 15 min prior to the enzymatic debridement procedure [13].

In oral administration of bromelain, tachyarrhythmias, decreased platelet aggregation, decreased fibrinogen plasma levels, and moderate prolongation of thromboplastin time and prothrombin time have been reported in the literature. It is recommended to apply NexoBrid™ at a maximum of 15% TBSA for a single application, because the bromelain-enriched proteolytic mixture is systemically absorbed. If more extensive debridement is required, the bromelain-based enzymatic debridement must be applied repeatedly in portions [13].

The bromelain-based agent was not tested in patients with unstable vital functions during pre-registration studies. Up to now we did not find any relevant publications confirming the safe use of bromelain-based agent in unstable burned patients. Our experience, as well as the basic principles of the bromelain-based enzymatic debridement did prove that the enzymatic debridement should become a treatment of choice in patients with deep burns where surgical debridement in general anaesthesia could present a high risk for them.

## 4 Conclusion

Thanks to the confirmed high selectivity, the bromelain-based enzymatic debridement could become a method of choice, especially in the treatment of deep facial burns. The new enzymatic debridement supposes to be a safer alternative to the surgical necrosectomy in unstable patients with deep burns. Moreover, the enzymatic debridement can be used as a time-saving tool in the management of high-risk deeply burned patients who require surgical intervention.

## Declaration of Competing Interest

All authors have no conflict of interest to declare.

## Sources of funding

This work was supported by Ministry of Health of Slovak Republicunder the project No. 2019/17-LFUK-5 (301/2019). The funding source has no involvement in study realization and interpretation.

## Ethical approval

Case reports are exempt from ethical approval at our institution.

## Consent

Written informed consent was obtained from the patient for publication of this case report and accompanying images. A copy of the written consent is available for review by the Editor-in-Chief of this journal on request.

## Author contribution

**Ferancikova Nikola:** Investigation, Data curation, Writing- Original draft preparation

**Bukovcan Peter:** Methodology, Validation, Formal analysis

**Sarkozyova Nina:** Investigation, Data Curation

**Dragunova Jana:** Resources, Funding acquisition

**Cucorova Valeria:** Resources, Funding acquisition

**Koller Jan:** Conceptualization, Writing - Review & Editing, Supervision

## Registration of research studies

Not applicable.

## Guarantor

Prof. Koller Jan PhD., M.D.

## Provenance and peer review

Not commissioned, externally peer-reviewed
